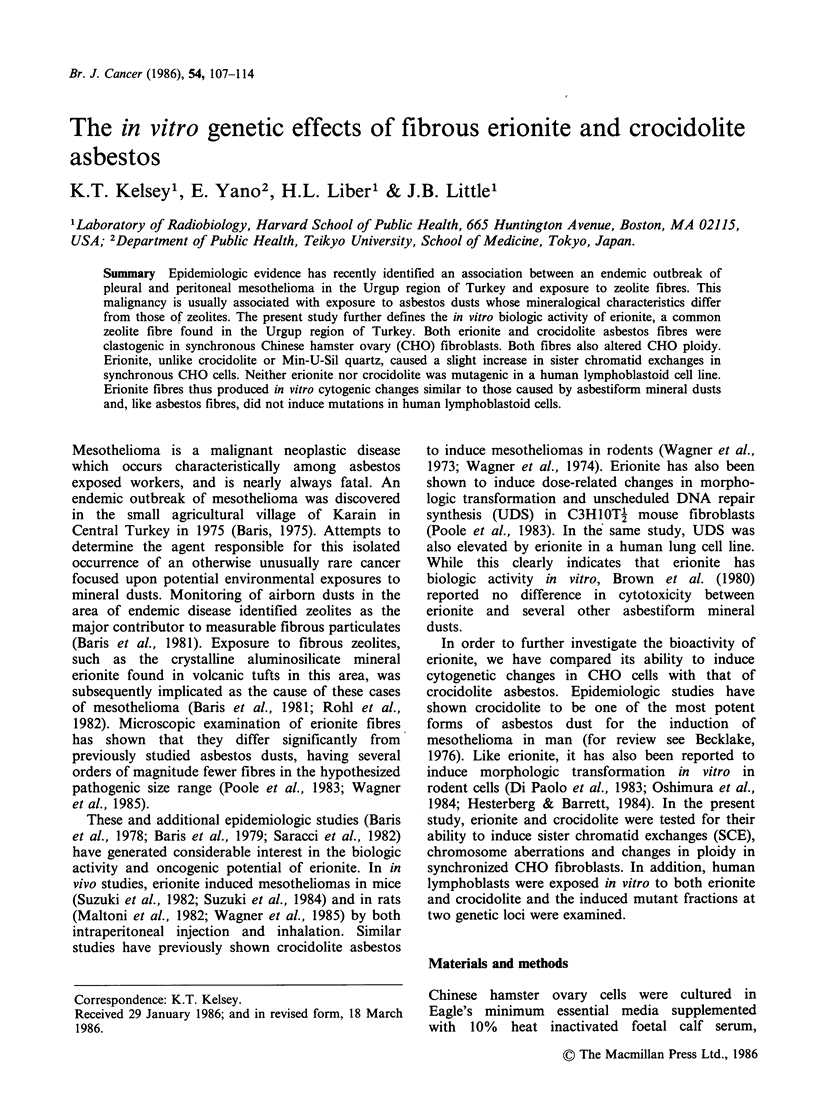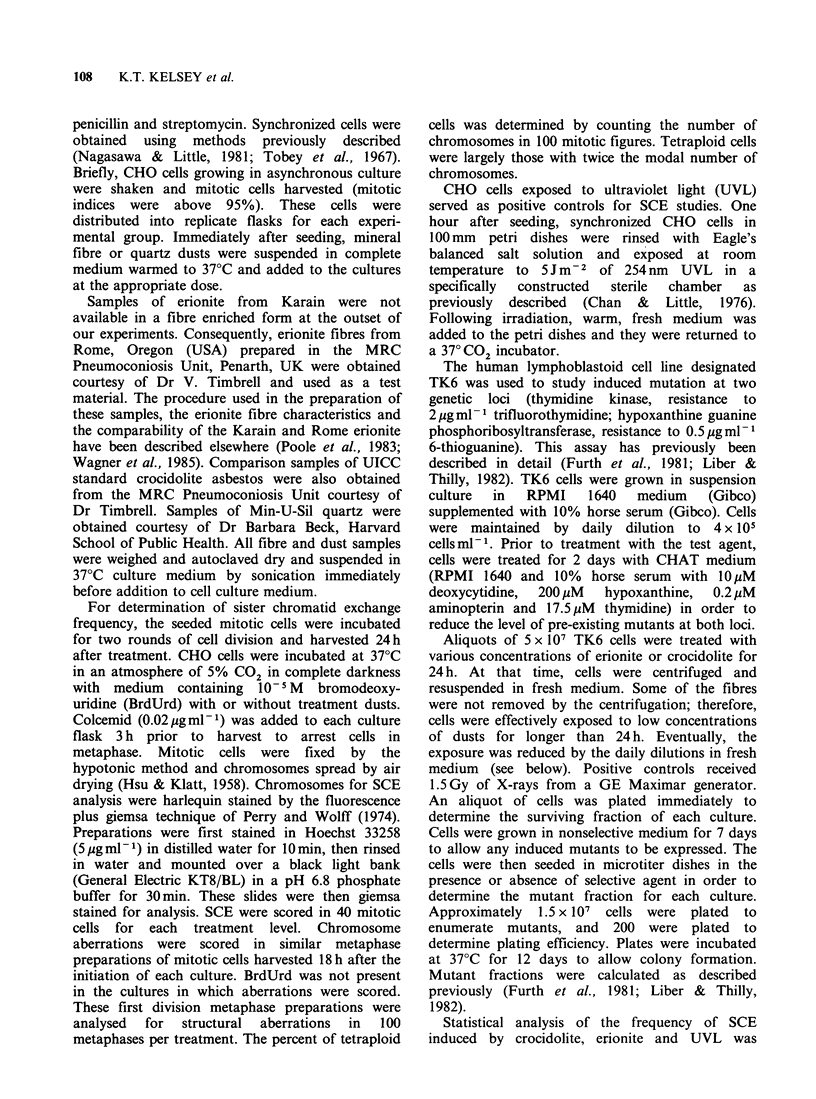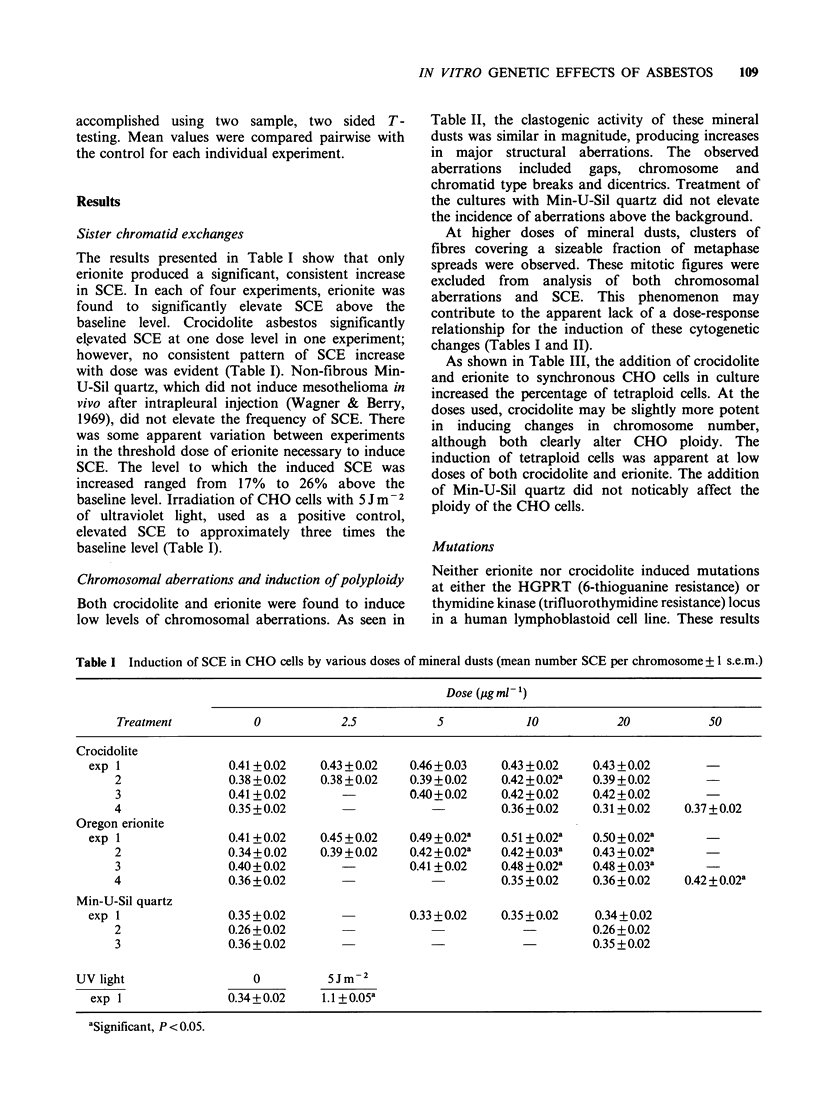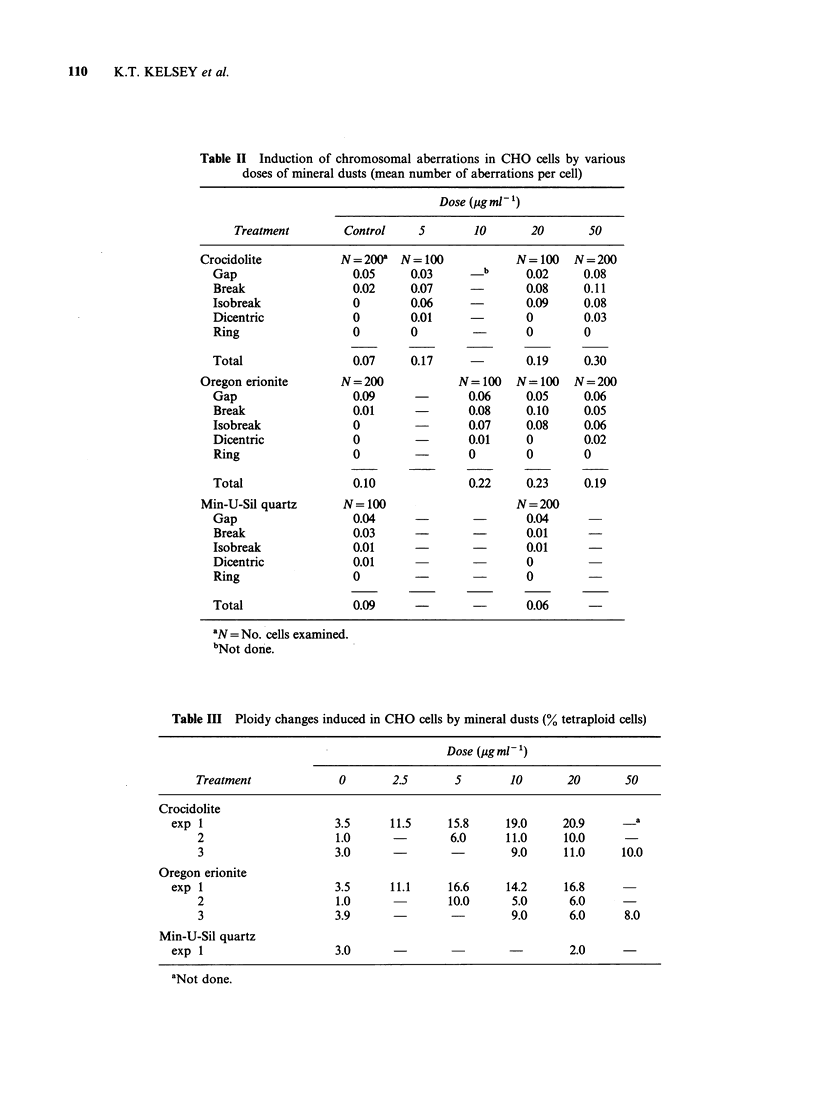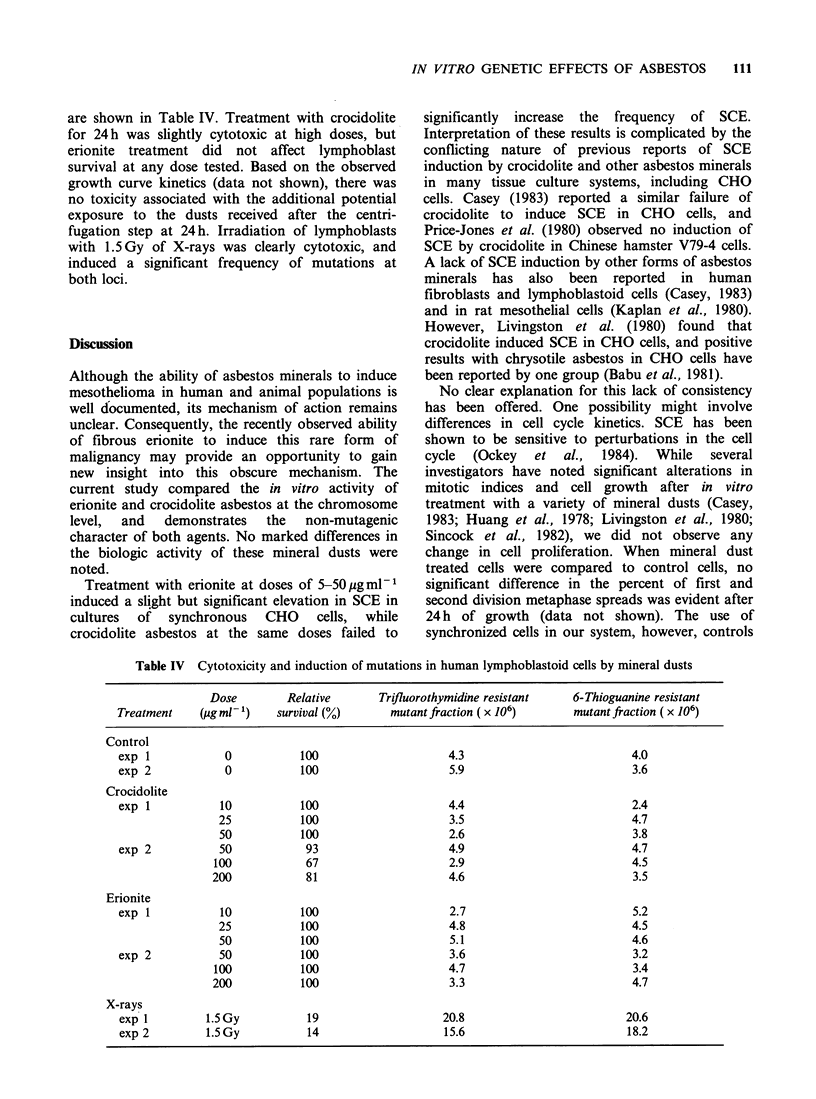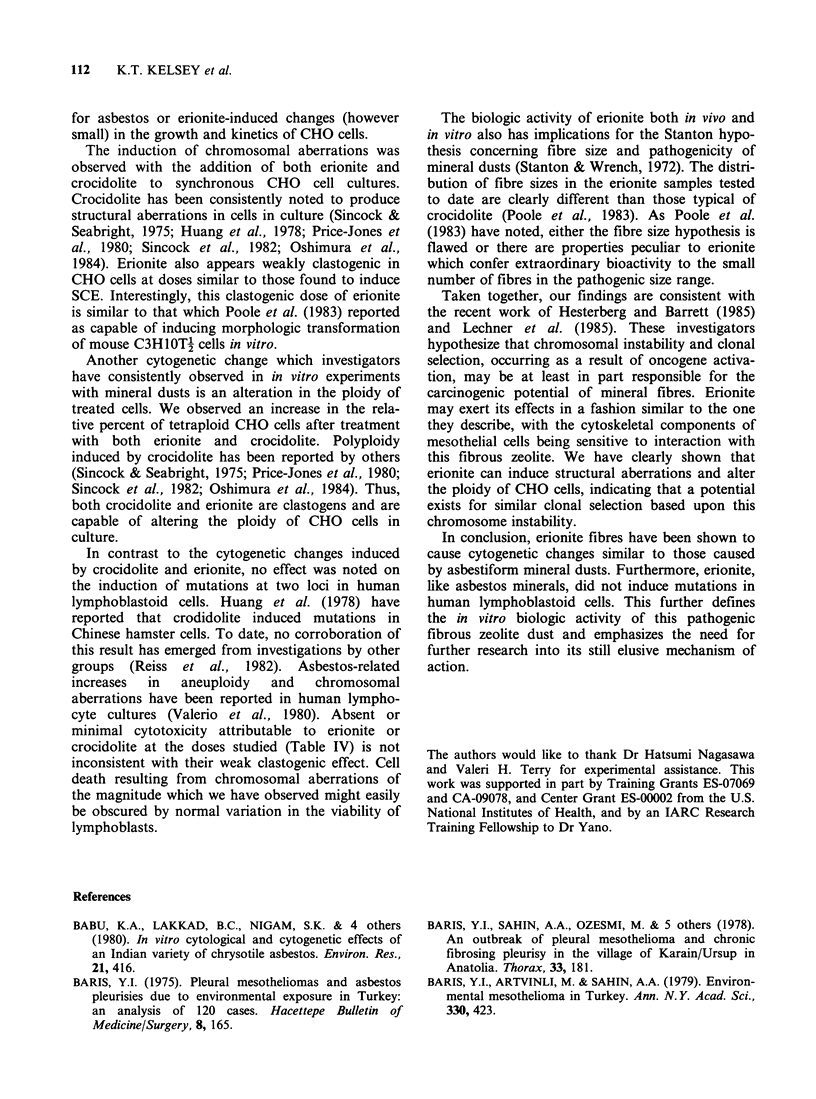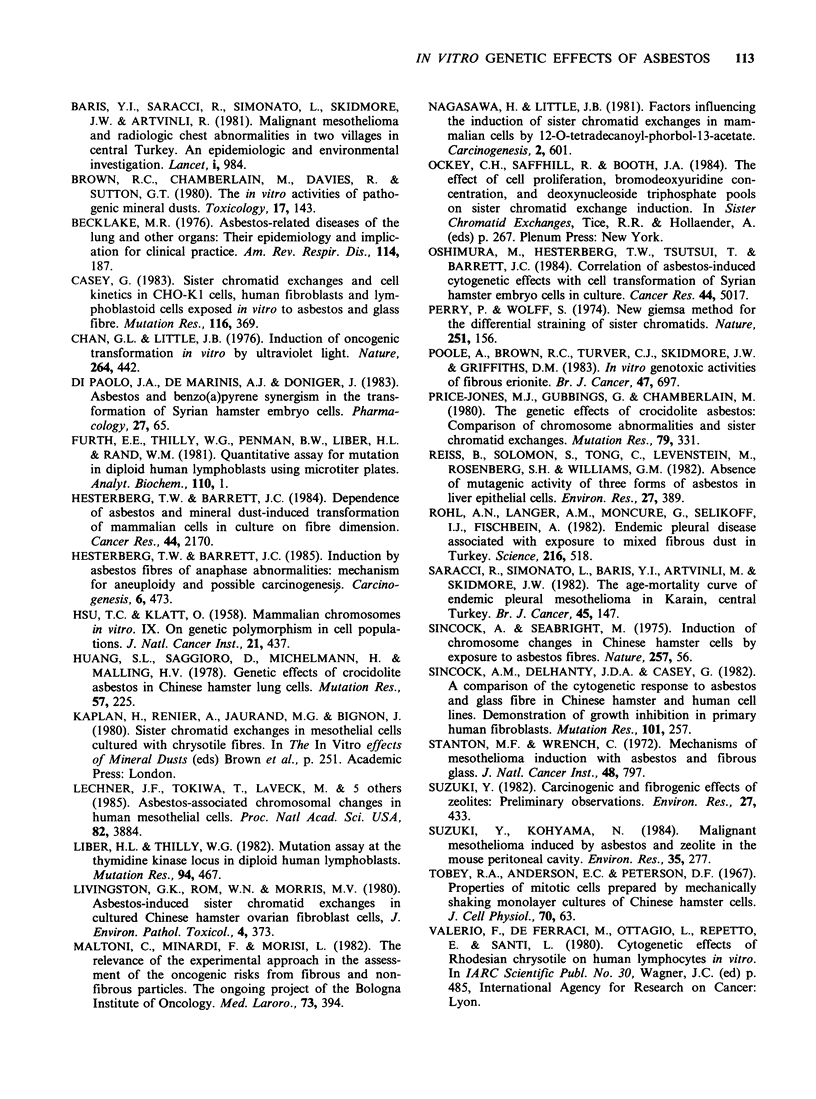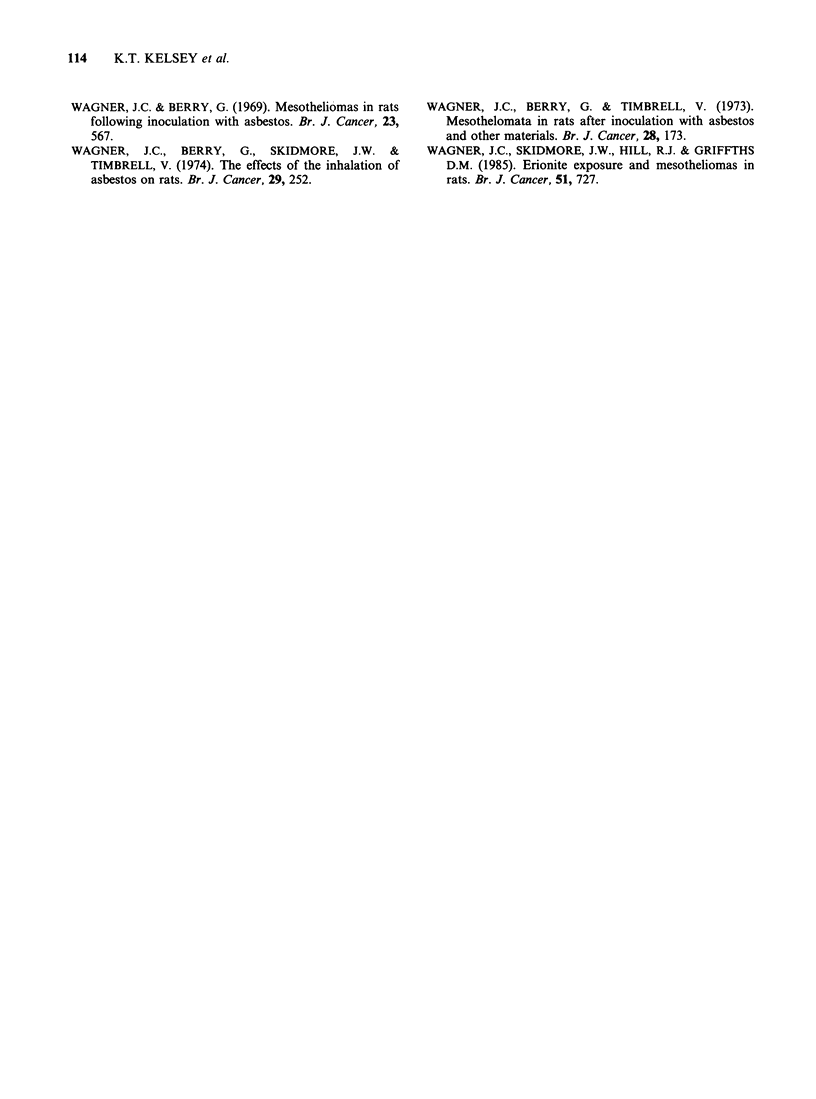# The in vitro genetic effects of fibrous erionite and crocidolite asbestos.

**DOI:** 10.1038/bjc.1986.158

**Published:** 1986-07

**Authors:** K. T. Kelsey, E. Yano, H. L. Liber, J. B. Little

## Abstract

Epidemiologic evidence has recently identified an association between an endemic outbreak of pleural and peritoneal mesothelioma in the Urgup region of Turkey and exposure to zeolite fibres. This malignancy is usually associated with exposure to asbestos dusts whose mineralogical characteristics differ from those of zeolites. The present study further defines the in vitro biologic activity of erionite, a common zeolite fibre found in the Urgup region of Turkey. Both erionite and crocidolite asbestos fibres were clastogenic in synchronous Chinese hamster ovary (CHO) fibroblasts. Both fibres also altered CHO ploidy. Erionite, unlike crocidolite or Min-U-Sil quartz, caused a slight increase in sister chromatid exchanges in synchronous CHO cells. Neither erionite nor crocidolite was mutagenic in a human lymphoblastoid cell line. Erionite fibres thus produced in vitro cytogenic changes similar to those caused by asbestiform mineral dusts and, like asbestos fibres, did not induce mutations in human lymphoblastoid cells.


*Br. J. Cancer* (1986), **54**, 107–114

### The *in vitro* genetic effects of fibrous erionite and crocidolite asbestos

K.T. Kelsey[1], E. Yano[2], H.L. Liber[1] & J.B. Little[1]

[1]*Laboratory of Radiobiology, Harvard School of Public Health, 665 Huntington Avenue, Boston, MA 02115, USA;* [2]*Department of Public Health, Teikyo University, School of Medicine, Tokyo, Japan.*

**Summary** Epidemiologic evidence has recently identified an association between an endemic outbreak of pleural and peritoneal mesothelioma in the Urgup region of Turkey and exposure to zeolite fibres. This malignancy is usually associated with exposure to asbestos dusts whose mineralogical characteristics differ from those of zeolites. The present study further defines the *in vitro* biologic activity of erionite, a common zeolite fibre found in the Urgup region of Turkey. Both erionite and crocidolite asbestos fibres were clastogenic in synchronous Chinese hamster ovary (CHO) fibroblasts. Both fibres also altered CHO ploidy. Erionite, unlike crocidolite or Min-U-Sil quartz, caused a slight increase in sister chromatid exchanges in synchronous CHO cells. Neither erionite nor crocidolite was mutagenic in a human lymphoblastoid cell line. Erionite fibres thus produced *in vitro* cytogenic changes similar to those caused by asbestiform mineral dusts and, like asbestos fibres, did not induce mutations in human lymphoblastoid cells.

Mesothelioma is a malignant neoplastic disease which occurs characteristically among asbestos exposed workers, and is nearly always fatal. An endemic outbreak of mesothelioma was discovered in the small agricultural village of Karain in Central Turkey in 1975 (Baris, 1975). Attempts to determine the agent responsible for this isolated occurrence of an otherwise unusually rare cancer focused upon potential environmental exposures to mineral dusts. Monitoring of airborn dusts in the area of endemic disease identified zeolites as the major contributor to measurable fibrous particulates (Baris *et al.*, 1981). Exposure to fibrous zeolites, such as the crystalline aluminosilicate mineral erionite found in volcanic tufts in this area, was subsequently implicated as the cause of these cases of mesothelioma (Baris *et al.*, 1981; Rohl *et al.*, 1982). Microscopic examination of erionite fibres has shown that they differ significantly from previously studied asbestos dusts, having several orders of magnitude fewer fibres in the hypothesized pathogenic size range (Poole *et al.*, 1983; Wagner *et al.*, 1985).

These and additional epidemiologic studies (Baris *et al.*, 1978; Baris *et al.*, 1979; Saracci *et al.*, 1982) have generated considerable interest in the biologic activity and oncogenic potential of erionite. In *in vivo* studies, erionite induced mesotheliomas in mice (Suzuki *et al.*, 1982; Suzuki *et al.*, 1984) and in rats (Maltoni *et al.*, 1982; Wagner *et al.*, 1985) by both intraperitoneal injection and inhalation. Similar studies have previously shown crocidolite asbestos to induce mesotheliomas in rodents (Wagner *et al.*, 1973; Wagner *et al.*, 1974). Erionite has also been shown to induce dose-related changes in morphologic transformation and unscheduled DNA repair synthesis (UDS) in C3H10T$\frac{1}{2}$ mouse fibroblasts (Poole *et al.*, 1983). In the same study, UDS was also elevated by erionite in a human lung cell line. While this clearly indicates that erionite has biologic activity *in vitro*, Brown *et al.* (1980) reported no difference in cytotoxicity between erionite and several other asbestiform mineral dusts.

In order to further investigate the bioactivity of erionite, we have compared its ability to induce cytogenetic changes in CHO cells with that of crocidolite asbestos. Epidemiologic studies have shown crocidolite to be one of the most potent forms of asbestos dust for the induction of mesothelioma in man (for review see Becklake, 1976). Like erionite, it has also been reported to induce morphologic transformation *in vitro* in rodent cells (Di Paolo *et al.*, 1983; Oshimura *et al.*, 1984; Hesterberg & Barrett, 1984). In the present study, erionite and crocidolite were tested for their ability to induce sister chromatid exchanges (SCE), chromosome aberrations and changes in ploidy in synchronized CHO fibroblasts. In addition, human lymphoblasts were exposed *in vitro* to both erionite and crocidolite and the induced mutant fractions at two genetic loci were examined.

### Materials and methods

Chinese hamster ovary cells were cultured in Eagle's minimum essential media supplemented with 10% heat inactivated foetal calf serum,

Correspondence: K.T. Kelsey.
Received 29 January 1986; and in revised form, 18 March 1986.

© The Macmillan Press Ltd., 1986

penicillin and streptomycin. Synchronized cells were obtained using methods previously described (Nagasawa & Little, 1981; Tobey *et al.*, 1967). Briefly, CHO cells growing in asynchronous culture were shaken and mitotic cells harvested (mitotic indices were above 95%). These cells were distributed into replicate flasks for each experimental group. Immediately after seeding, mineral fibre or quartz dusts were suspended in complete medium warmed to 37°C and added to the cultures at the appropriate dose.

Samples of erionite from Karain were not available in a fibre enriched form at the outset of our experiments. Consequently, erionite fibres from Rome, Oregon (USA) prepared in the MRC Pneumoconiosis Unit, Penarth, UK were obtained courtesy of Dr V. Timbrell and used as a test material. The procedure used in the preparation of these samples, the erionite fibre characteristics and the comparability of the Karain and Rome erionite have been described elsewhere (Poole *et al.*, 1983; Wagner *et al.*, 1985). Comparison samples of UICC standard crocidolite asbestos were also obtained from the MRC Pneumoconiosis Unit courtesy of Dr Timbrell. Samples of Min-U-Sil quartz were obtained courtesy of Dr Barbara Beck, Harvard School of Public Health. All fibre and dust samples were weighed and autoclaved dry and suspended in 37°C culture medium by sonication immediately before addition to cell culture medium.

For determination of sister chromatid exchange frequency, the seeded mitotic cells were incubated for two rounds of cell division and harvested 24 h after treatment. CHO cells were incubated at 37°C in an atmosphere of 5% $CO_2$ in complete darkness with medium containing $10^{-5}$ M bromodeoxyuridine (BrdUrd) with or without treatment dusts. Colcemid (0.02 $\mu g\,ml^{-1}$) was added to each culture flask 3 h prior to harvest to arrest cells in metaphase. Mitotic cells were fixed by the hypotonic method and chromosomes spread by air drying (Hsu & Klatt, 1958). Chromosomes for SCE analysis were harlequin stained by the fluorescence plus giemsa technique of Perry and Wolff (1974). Preparations were first stained in Hoechst 33258 (5 $\mu g\,ml^{-1}$) in distilled water for 10 min, then rinsed in water and mounted over a black light bank (General Electric KT8/BL) in a pH 6.8 phosphate buffer for 30 min. These slides were then giemsa stained for analysis. SCE were scored in 40 mitotic cells for each treatment level. Chromosome aberrations were scored in similar metaphase preparations of mitotic cells harvested 18 h after the initiation of each culture. BrdUrd was not present in the cultures in which aberrations were scored. These first division metaphase preparations were analysed for structural aberrations in 100 metaphases per treatment. The percent of tetraploid cells was determined by counting the number of chromosomes in 100 mitotic figures. Tetraploid cells were largely those with twice the modal number of chromosomes.

CHO cells exposed to ultraviolet light (UVL) served as positive controls for SCE studies. One hour after seeding, synchronized CHO cells in 100 mm petri dishes were rinsed with Eagle's balanced salt solution and exposed at room temperature to 5 $J\,m^{-2}$ of 254 nm UVL in a specifically constructed sterile chamber as previously described (Chan & Little, 1976). Following irradiation, warm, fresh medium was added to the petri dishes and they were returned to a 37° $CO_2$ incubator.

The human lymphoblastoid cell line designated TK6 was used to study induced mutation at two genetic loci (thymidine kinase, resistance to 2 $\mu g\,ml^{-1}$ trifluorothymidine; hypoxanthine guanine phosphoribosyltransferase, resistance to 0.5 $\mu g\,ml^{-1}$ 6-thioguanine). This assay has previously been described in detail (Furth *et al.*, 1981; Liber & Thilly, 1982). TK6 cells were grown in suspension culture in RPMI 1640 medium (Gibco) supplemented with 10% horse serum (Gibco). Cells were maintained by daily dilution to $4 \times 10^5$ cells $ml^{-1}$. Prior to treatment with the test agent, cells were treated for 2 days with CHAT medium (RPMI 1640 and 10% horse serum with 10 $\mu$M deoxycytidine, 200 $\mu$M hypoxanthine, 0.2 $\mu$M aminopterin and 17.5 $\mu$M thymidine) in order to reduce the level of pre-existing mutants at both loci.

Aliquots of $5 \times 10^7$ TK6 cells were treated with various concentrations of erionite or crocidolite for 24 h. At that time, cells were centrifuged and resuspended in fresh medium. Some of the fibres were not removed by the centrifugation; therefore, cells were effectively exposed to low concentrations of dusts for longer than 24 h. Eventually, the exposure was reduced by the daily dilutions in fresh medium (see below). Positive controls received 1.5 Gy of X-rays from a GE Maximar generator. An aliquot of cells was plated immediately to determine the surviving fraction of each culture. Cells were grown in nonselective medium for 7 days to allow any induced mutants to be expressed. The cells were then seeded in microtiter dishes in the presence or absence of selective agent in order to determine the mutant fraction for each culture. Approximately $1.5 \times 10^7$ cells were plated to enumerate mutants, and 200 were plated to determine plating efficiency. Plates were incubated at 37°C for 12 days to allow colony formation. Mutant fractions were calculated as described previously (Furth *et al.*, 1981; Liber & Thilly, 1982).

Statistical analysis of the frequency of SCE induced by crocidolite, erionite and UVL was accomplished using two sample, two sided *T*-testing. Mean values were compared pairwise with the control for each individual experiment.

### Results

### *Sister chromatid exchanges*

The results presented in Table I show that only erionite produced a significant, consistent increase in SCE. In each of four experiments, erionite was found to significantly elevate SCE above the baseline level. Crocidolite asbestos significantly elevated SCE at one dose level in one experiment; however, no consistent pattern of SCE increase with dose was evident (Table I). Non-fibrous Min-U-Sil quartz, which did not induce mesothelioma *in vivo* after intrapleural injection (Wagner & Berry, 1969), did not elevate the frequency of SCE. There was some apparent variation between experiments in the threshold dose of erionite necessary to induce SCE. The level to which the induced SCE was increased ranged from 17% to 26% above the baseline level. Irradiation of CHO cells with 5 J $m^{-2}$ of ultraviolet light, used as a positive control, elevated SCE to approximately three times the baseline level (Table I).

### *Chromosomal aberrations and induction of polyploidy*

Both crocidolite and erionite were found to induce low levels of chromosomal aberrations. As seen in Table II, the clastogenic activity of these mineral dusts was similar in magnitude, producing increases in major structural aberrations. The observed aberrations included gaps, chromosome and chromatid type breaks and dicentrics. Treatment of the cultures with Min-U-Sil quartz did not elevate the incidence of aberrations above the background.

At higher doses of mineral dusts, clusters of fibres covering a sizeable fraction of metaphase spreads were observed. These mitotic figures were excluded from analysis of both chromosomal aberrations and SCE. This phenomenon may contribute to the apparent lack of a dose-response relationship for the induction of these cytogenetic changes (Tables I and II).

As shown in Table III, the addition of crocidolite and erionite to synchronous CHO cells in culture increased the percentage of tetraploid cells. At the doses used, crocidolite may be slightly more potent in inducing changes in chromosome number, although both clearly alter CHO ploidy. The induction of tetraploid cells was apparent at low doses of both crocidolite and erionite. The addition of Min-U-Sil quartz did not noticably affect the ploidy of the CHO cells.

### *Mutations*

Neither erionite nor crocidolite induced mutations at either the HGPRT (6-thioguanine resistance) or thymidine kinase (trifluorothymidine resistance) locus in a human lymphoblastoid cell line. These results

**Table I** Induction of SCE in CHO cells by various doses of mineral dusts (mean number SCE per chromosome ± 1 s.e.m.)

| | *Dose* ($\mu g\ ml^{-1}$) | | | | | |
|---|---|---|---|---|---|---|
| *Treatment* | *0* | *2.5* | *5* | *10* | *20* | *50* |
| Crocidolite | | | | | | |
| exp 1 | 0.41±0.02 | 0.43±0.02 | 0.46±0.03 | 0.43±0.02 | 0.43±0.02 | — |
| 2 | 0.38±0.02 | 0.38±0.02 | 0.39±0.02 | 0.42±0.02[a] | 0.39±0.02 | — |
| 3 | 0.41±0.02 | — | 0.40±0.02 | 0.42±0.02 | 0.42±0.02 | — |
| 4 | 0.35±0.02 | — | — | 0.36±0.02 | 0.31±0.02 | 0.37±0.02 |
| Oregon erionite | | | | | | |
| exp 1 | 0.41±0.02 | 0.45±0.02 | 0.49±0.02[a] | 0.51±0.02[a] | 0.50±0.02[a] | — |
| 2 | 0.34±0.02 | 0.39±0.02 | 0.42±0.02[a] | 0.42±0.03[a] | 0.43±0.02[a] | — |
| 3 | 0.40±0.02 | — | 0.41±0.02 | 0.48±0.02[a] | 0.48±0.03[a] | — |
| 4 | 0.36±0.02 | — | — | 0.35±0.02 | 0.36±0.02 | 0.42±0.02[a] |
| Min-U-Sil quartz | | | | | | |
| exp 1 | 0.35±0.02 | — | 0.33±0.02 | 0.35±0.02 | 0.34±0.02 | |
| 2 | 0.26±0.02 | — | — | — | 0.26±0.02 | |
| 3 | 0.36±0.02 | — | — | — | 0.35±0.02 | |
| UV light | 0 | 5 J $m^{-2}$ | | | | |
| exp 1 | 0.34±0.02 | 1.1±0.05[a] | | | | |

[a]Significant, $P<0.05$.

**Table II** Induction of chromosomal aberrations in CHO cells by various doses of mineral dusts (mean number of aberrations per cell)

| | *Dose* ($\mu g\,ml^{-1}$) | | | | |
|---|---|---|---|---|---|
| *Treatment* | *Control* | *5* | *10* | *20* | *50* |
| Crocidolite | $N=200$[a] | $N=100$ | | $N=100$ | $N=200$ |
| Gap | 0.05 | 0.03 | —[b] | 0.02 | 0.08 |
| Break | 0.02 | 0.07 | — | 0.08 | 0.11 |
| Isobreak | 0 | 0.06 | — | 0.09 | 0.08 |
| Dicentric | 0 | 0.01 | — | 0 | 0.03 |
| Ring | 0 | 0 | — | 0 | 0 |
| Total | 0.07 | 0.17 | — | 0.19 | 0.30 |
| Oregon erionite | $N=200$ | | $N=100$ | $N=100$ | $N=200$ |
| Gap | 0.09 | — | 0.06 | 0.05 | 0.06 |
| Break | 0.01 | — | 0.08 | 0.10 | 0.05 |
| Isobreak | 0 | — | 0.07 | 0.08 | 0.06 |
| Dicentric | 0 | — | 0.01 | 0 | 0.02 |
| Ring | 0 | — | 0 | 0 | 0 |
| Total | 0.10 | | 0.22 | 0.23 | 0.19 |
| Min-U-Sil quartz | $N=100$ | | | $N=200$ | |
| Gap | 0.04 | — | — | 0.04 | — |
| Break | 0.03 | — | — | 0.01 | — |
| Isobreak | 0.01 | — | — | 0.01 | — |
| Dicentric | 0.01 | — | — | 0 | — |
| Ring | 0 | — | — | 0 | — |
| Total | 0.09 | — | — | 0.06 | — |

[a]$N$ = No. cells examined.
[b]Not done.

**Table III** Ploidy changes induced in CHO cells by mineral dusts (% tetraploid cells)

| | *Dose* ($\mu g\,ml^{-1}$) | | | | | |
|---|---|---|---|---|---|---|
| *Treatment* | *0* | *2.5* | *5* | *10* | *20* | *50* |
| Crocidolite | | | | | | |
| exp 1 | 3.5 | 11.5 | 15.8 | 19.0 | 20.9 | —[a] |
| 2 | 1.0 | — | 6.0 | 11.0 | 10.0 | — |
| 3 | 3.0 | — | — | 9.0 | 11.0 | 10.0 |
| Oregon erionite | | | | | | |
| exp 1 | 3.5 | 11.1 | 16.6 | 14.2 | 16.8 | — |
| 2 | 1.0 | — | 10.0 | 5.0 | 6.0 | — |
| 3 | 3.9 | — | — | 9.0 | 6.0 | 8.0 |
| Min-U-Sil quartz | | | | | | |
| exp 1 | 3.0 | — | — | — | 2.0 | — |

[a]Not done.

are shown in Table IV. Treatment with crocidolite for 24 h was slightly cytotoxic at high doses, but erionite treatment did not affect lymphoblast survival at any dose tested. Based on the observed growth curve kinetics (data not shown), there was no toxicity associated with the additional potential exposure to the dusts received after the centrifugation step at 24 h. Irradiation of lymphoblasts with 1.5 Gy of X-rays was clearly cytotoxic, and induced a significant frequency of mutations at both loci.

### Discussion

Although the ability of asbestos minerals to induce mesothelioma in human and animal populations is well documented, its mechanism of action remains unclear. Consequently, the recently observed ability of fibrous erionite to induce this rare form of malignancy may provide an opportunity to gain new insight into this obscure mechanism. The current study compared the *in vitro* activity of erionite and crocidolite asbestos at the chromosome level, and demonstrates the non-mutagenic character of both agents. No marked differences in the biologic activity of these mineral dusts were noted.

Treatment with erionite at doses of 5–50 $\mu g\,ml^{-1}$ induced a slight but significant elevation in SCE in cultures of synchronous CHO cells, while crocidolite asbestos at the same doses failed to significantly increase the frequency of SCE. Interpretation of these results is complicated by the conflicting nature of previous reports of SCE induction by crocidolite and other asbestos minerals in many tissue culture systems, including CHO cells. Casey (1983) reported a similar failure of crocidolite to induce SCE in CHO cells, and Price-Jones *et al.* (1980) observed no induction of SCE by crocidolite in Chinese hamster V79-4 cells. A lack of SCE induction by other forms of asbestos minerals has also been reported in human fibroblasts and lymphoblastoid cells (Casey, 1983) and in rat mesothelial cells (Kaplan *et al.*, 1980). However, Livingston *et al.* (1980) found that crocidolite induced SCE in CHO cells, and positive results with chrysotile asbestos in CHO cells have been reported by one group (Babu *et al.*, 1981).

No clear explanation for this lack of consistency has been offered. One possibility might involve differences in cell cycle kinetics. SCE has been shown to be sensitive to perturbations in the cell cycle (Ockey *et al.*, 1984). While several investigators have noted significant alterations in mitotic indices and cell growth after *in vitro* treatment with a variety of mineral dusts (Casey, 1983; Huang *et al.*, 1978; Livingston *et al.*, 1980; Sincock *et al.*, 1982), we did not observe any change in cell proliferation. When mineral dust treated cells were compared to control cells, no significant difference in the percent of first and second division metaphase spreads was evident after 24 h of growth (data not shown). The use of synchronized cells in our system, however, controls

**Table IV** Cytotoxicity and induction of mutations in human lymphoblastoid cells by mineral dusts

| *Treatment* | *Dose* ($\mu g\,ml^{-1}$) | *Relative survival* (%) | *Trifluorothymidine resistant mutant fraction* ( $\times 10^6$) | *6-Thioguanine resistant mutant fraction* ( $\times 10^6$) |
|---|---|---|---|---|
| Control | | | | |
| exp 1 | 0 | 100 | 4.3 | 4.0 |
| exp 2 | 0 | 100 | 5.9 | 3.6 |
| Crocidolite | | | | |
| exp 1 | 10 | 100 | 4.4 | 2.4 |
| | 25 | 100 | 3.5 | 4.7 |
| | 50 | 100 | 2.6 | 3.8 |
| exp 2 | 50 | 93 | 4.9 | 4.7 |
| | 100 | 67 | 2.9 | 4.5 |
| | 200 | 81 | 4.6 | 3.5 |
| Erionite | | | | |
| exp 1 | 10 | 100 | 2.7 | 5.2 |
| | 25 | 100 | 4.8 | 4.5 |
| | 50 | 100 | 5.1 | 4.6 |
| exp 2 | 50 | 100 | 3.6 | 3.2 |
| | 100 | 100 | 4.7 | 3.4 |
| | 200 | 100 | 3.3 | 4.7 |
| X-rays | | | | |
| exp 1 | 1.5 Gy | 19 | 20.8 | 20.6 |
| exp 2 | 1.5 Gy | 14 | 15.6 | 18.2 |

for asbestos or erionite-induced changes (however small) in the growth and kinetics of CHO cells.

The induction of chromosomal aberrations was observed with the addition of both erionite and crocidolite to synchronous CHO cell cultures. Crocidolite has been consistently noted to produce structural aberrations in cells in culture (Sincock & Seabright, 1975; Huang *et al.*, 1978; Price-Jones *et al.*, 1980; Sincock *et al.*, 1982; Oshimura *et al.*, 1984). Erionite also appears weakly clastogenic in CHO cells at doses similar to those found to induce SCE. Interestingly, this clastogenic dose of erionite is similar to that which Poole *et al.* (1983) reported as capable of inducing morphologic transformation of mouse C3H10T$\frac{1}{2}$ cells *in vitro*.

Another cytogenetic change which investigators have consistently observed in *in vitro* experiments with mineral dusts is an alteration in the ploidy of treated cells. We observed an increase in the relative percent of tetraploid CHO cells after treatment with both erionite and crocidolite. Polyploidy induced by crocidolite has been reported by others (Sincock & Seabright, 1975; Price-Jones *et al.*, 1980; Sincock *et al.*, 1982; Oshimura *et al.*, 1984). Thus, both crocidolite and erionite are clastogens and are capable of altering the ploidy of CHO cells in culture.

In contrast to the cytogenetic changes induced by crocidolite and erionite, no effect was noted on the induction of mutations at two loci in human lymphoblastoid cells. Huang *et al.* (1978) have reported that crodidolite induced mutations in Chinese hamster cells. To date, no corroboration of this result has emerged from investigations by other groups (Reiss *et al.*, 1982). Asbestos-related increases in aneuploidy and chromosomal aberrations have been reported in human lymphocyte cultures (Valerio *et al.*, 1980). Absent or minimal cytotoxicity attributable to erionite or crocidolite at the doses studied (Table IV) is not inconsistent with their weak clastogenic effect. Cell death resulting from chromosomal aberrations of the magnitude which we have observed might easily be obscured by normal variation in the viability of lymphoblasts.

The biologic activity of erionite both *in vivo* and *in vitro* also has implications for the Stanton hypothesis concerning fibre size and pathogenicity of mineral dusts (Stanton & Wrench, 1972). The distribution of fibre sizes in the erionite samples tested to date are clearly different than those typical of crocidolite (Poole *et al.*, 1983). As Poole *et al.* (1983) have noted, either the fibre size hypothesis is flawed or there are properties peculiar to erionite which confer extraordinary bioactivity to the small number of fibres in the pathogenic size range.

Taken together, our findings are consistent with the recent work of Hesterberg and Barrett (1985) and Lechner *et al.* (1985). These investigators hypothesize that chromosomal instability and clonal selection, occurring as a result of oncogene activation, may be at least in part responsible for the carcinogenic potential of mineral fibres. Erionite may exert its effects in a fashion similar to the one they describe, with the cytoskeletal components of mesothelial cells being sensitive to interaction with this fibrous zeolite. We have clearly shown that erionite can induce structural aberrations and alter the ploidy of CHO cells, indicating that a potential exists for similar clonal selection based upon this chromosome instability.

In conclusion, erionite fibres have been shown to cause cytogenetic changes similar to those caused by asbestiform mineral dusts. Furthermore, erionite, like asbestos minerals, did not induce mutations in human lymphoblastoid cells. This further defines the *in vitro* biologic activity of this pathogenic fibrous zeolite dust and emphasizes the need for further research into its still elusive mechanism of action.

The authors would like to thank Dr Hatsumi Nagasawa and Valeri H. Terry for experimental assistance. This work was supported in part by Training Grants ES-07069 and CA-09078, and Center Grant ES-00002 from the U.S. National Institutes of Health, and by an IARC Research Training Fellowship to Dr Yano.

### References

BABU, K.A., LAKKAD, B.C., NIGAM, S.K. & 4 others (1980). *In vitro* cytological and cytogenetic effects of an Indian variety of chrysotile asbestos. *Environ. Res.*, **21**, 416.

BARIS, Y.I. (1975). Pleural mesotheliomas and asbestos pleurisies due to environmental exposure in Turkey: an analysis of 120 cases. *Hacettepe Bulletin of Medicine/Surgery*, **8**, 165.

BARIS, Y.I., SAHIN, A.A., OZESMI, M. & 5 others (1978). An outbreak of pleural mesothelioma and chronic fibrosing pleurisy in the village of Karain/Ursup in Anatolia. *Thorax*, **33**, 181.

BARIS, Y.I., ARTVINLI, M. & SAHIN, A.A. (1979). Environmental mesothelioma in Turkey. *Ann. N.Y. Acad. Sci.*, **330**, 423.

BARIS, Y.I., SARACCI, R., SIMONATO, L., SKIDMORE, J.W. & ARTVINLI, R. (1981). Malignant mesothelioma and radiologic chest abnormalities in two villages in central Turkey. An epidemiologic and environmental investigation. *Lancet*, **i**, 984.

BROWN, R.C., CHAMBERLAIN, M., DAVIES, R. & SUTTON, G.T. (1980). The *in vitro* activities of pathogenic mineral dusts. *Toxicology*, **17**, 143.

BECKLAKE, M.R. (1976). Asbestos-related diseases of the lung and other organs: Their epidemiology and implication for clinical practice. *Am. Rev. Respir. Dis.*, **114**, 187.

CASEY, G. (1983). Sister chromatid exchanges and cell kinetics in CHO-K1 cells, human fibroblasts and lymphoblastoid cells exposed *in vitro* to asbestos and glass fibre. *Mutation Res.*, **116**, 369.

CHAN, G.L. & LITTLE, J.B. (1976). Induction of oncogenic transformation *in vitro* by ultraviolet light. *Nature*, **264**, 442.

DI PAOLO, J.A., DE MARINIS, A.J. & DONIGER, J. (1983). Asbestos and benzo(a)pyrene synergism in the transformation of Syrian hamster embryo cells. *Pharmacology*, **27**, 65.

FURTH, E.E., THILLY, W.G., PENMAN, B.W., LIBER, H.L. & RAND, W.M. (1981). Quantitative assay for mutation in diploid human lymphoblasts using microtiter plates. *Analyt. Biochem.*, **110**, 1.

HESTERBERG, T.W. & BARRETT, J.C. (1984). Dependence of asbestos and mineral dust-induced transformation of mammalian cells in culture on fibre dimension. *Cancer Res.*, **44**, 2170.

HESTERBERG, T.W. & BARRETT, J.C. (1985). Induction by asbestos fibres of anaphase abnormalities: mechanism for aneuploidy and possible carcinogenesis. *Carcinogenesis*, **6**, 473.

HSU, T.C. & KLATT, O. (1958). Mammalian chromosomes *in vitro*. IX. On genetic polymorphism in cell populations. *J. Natl. Cancer Inst.*, **21**, 437.

HUANG, S.L., SAGGIORO, D., MICHELMANN, H. & MALLING, H.V. (1978). Genetic effects of crocidolite asbestos in Chinese hamster lung cells. *Mutation Res.*, **57**, 225.

KAPLAN, H., RENIER, A., JAURAND, M.G. & BIGNON, J. (1980). Sister chromatid exchanges in mesothelial cells cultured with chrysotile fibres. In *The* In Vitro *effects of Mineral Dusts* (eds) Brown *et al.*, p. 251. Academic Press: London.

LECHNER, J.F., TOKIWA, T., LAVECK, M. & 5 others (1985). Asbestos-associated chromosomal changes in human mesothelial cells. *Proc. Natl Acad. Sci. USA*, **82**, 3884.

LIBER, H.L. & THILLY, W.G. (1982). Mutation assay at the thymidine kinase locus in diploid human lymphoblasts. *Mutation Res.*, **94**, 467.

LIVINGSTON, G.K., ROM, W.N. & MORRIS, M.V. (1980). Asbestos-induced sister chromatid exchanges in cultured Chinese hamster ovarian fibroblast cells, *J. Environ. Pathol. Toxicol.*, **4**, 373.

MALTONI, C., MINARDI, F. & MORISI, L. (1982). The relevance of the experimental approach in the assessment of the oncogenic risks from fibrous and nonfibrous particles. The ongoing project of the Bologna Institute of Oncology. *Med. Laroro.*, **73**, 394.

NAGASAWA, H. & LITTLE, J.B. (1981). Factors influencing the induction of sister chromatid exchanges in mammalian cells by 12-O-tetradecanoyl-phorbol-13-acetate. *Carcinogenesis*, **2**, 601.

OCKEY, C.H., SAFFHILL, R. & BOOTH, J.A. (1984). The effect of cell proliferation, bromodeoxyuridine concentration, and deoxynucleoside triphosphate pools on sister chromatid exchange induction. In *Sister Chromatid Exchanges*, Tice, R.R. & Hollaender, A. (eds) p. 267. Plenum Press: New York.

OSHIMURA, M., HESTERBERG, T.W., TSUTSUI, T. & BARRETT, J.C. (1984). Correlation of asbestos-induced cytogenetic effects with cell transformation of Syrian hamster embryo cells in culture. *Cancer Res.* **44**, 5017.

PERRY, P. & WOLFF, S. (1974). New giemsa method for the differential straining of sister chromatids. *Nature*, **251**, 156.

POOLE, A., BROWN, R.C., TURVER, C.J., SKIDMORE, J.W. & GRIFFITHS, D.M. (1983). *In vitro* genotoxic activities of fibrous erionite. *Br. J. Cancer*, **47**, 697.

PRICE-JONES, M.J., GUBBINGS, G. & CHAMBERLAIN, M. (1980). The genetic effects of crocidolite asbestos: Comparison of chromosome abnormalities and sister chromatid exchanges. *Mutation Res.*, **79**, 331.

REISS, B., SOLOMON, S., TONG, C., LEVENSTEIN, M., ROSENBERG, S.H. & WILLIAMS, G.M. (1982). Absence of mutagenic activity of three forms of asbestos in liver epithelial cells. *Environ. Res.*, **27**, 389.

ROHL, A.N., LANGER, A.M., MONCURE, G., SELIKOFF, I.J., FISCHBEIN, A. (1982). Endemic pleural disease associated with exposure to mixed fibrous dust in Turkey. *Science*, **216**, 518.

SARACCI, R., SIMONATO, L., BARIS, Y.I., ARTVINLI, M. & SKIDMORE, J.W. (1982). The age-mortality curve of endemic pleural mesothelioma in Karain, central Turkey. *Br. J. Cancer*, **45**, 147.

SINCOCK, A. & SEABRIGHT, M. (1975). Induction of chromosome changes in Chinese hamster cells by exposure to asbestos fibres. *Nature*, **257**, 56.

SINCOCK, A.M., DELHANTY, J.D.A. & CASEY, G. (1982). A comparison of the cytogenetic response to asbestos and glass fibre in Chinese hamster and human cell lines. Demonstration of growth inhibition in primary human fibroblasts. *Mutation Res.*, **101**, 257.

STANTON, M.F. & WRENCH, C. (1972). Mechanisms of mesothelioma induction with asbestos and fibrous glass. *J. Natl. Cancer Inst.*, **48**, 797.

SUZUKI, Y. (1982). Carcinogenic and fibrogenic effects of zeolites: Preliminary observations. *Environ. Res.*, **27**, 433.

SUZUKI, Y., KOHYAMA, N. (1984). Malignant mesothelioma induced by asbestos and zeolite in the mouse peritoneal cavity. *Environ. Res.*, **35**, 277.

TOBEY, R.A., ANDERSON, E.C. & PETERSON, D.F. (1967). Properties of mitotic cells prepared by mechanically shaking monolayer cultures of Chinese hamster cells. *J. Cell Physiol.*, **70**, 63.

VALERIO, F., DE FERRACI, M., OTTAGIO, L., REPETTO, E. & SANTI, L. (1980). Cytogenetic effects of Rhodesian chrysotile on human lymphocytes *in vitro*. In *IARC Scientific Publ. No. 30*, Wagner, J.C. (ed) p. 485, International Agency for Research on Cancer: Lyon.

WAGNER, J.C. & BERRY, G. (1969). Mesotheliomas in rats following inoculation with asbestos. *Br. J. Cancer*, **23**, 567.

WAGNER, J.C., BERRY, G., SKIDMORE, J.W. & TIMBRELL, V. (1974). The effects of the inhalation of asbestos on rats. *Br. J. Cancer*, **29**, 252.

WAGNER, J.C., BERRY, G. & TIMBRELL, V. (1973). Mesothelomata in rats after inoculation with asbestos and other materials. *Br. J. Cancer*, **28**, 173.

WAGNER, J.C., SKIDMORE, J.W., HILL, R.J. & GRIFFTHS D.M. (1985). Erionite exposure and mesotheliomas in rats. *Br. J. Cancer*, **51**, 727.